# Impact of the bromination of carbazole-based D–π–A organic dyes on their optical and electrochemical properties and visible-light-driven hydrogen evolution†

**DOI:** 10.1039/d3ra02785f

**Published:** 2023-07-04

**Authors:** Zhangli Hu, Jiamin Kuang, Wenmo Fu, Longxin Hu, Hua Lai, Huanian Zhang, Xing Liu

**Affiliations:** a College of Chemistry Materials, Hengyang Normal University Hengyang 421008 China laixhua163@163.com; b Hunan Provincial Key Laboratory of Functional Metal-Organic Compounds Hengyang 421008 China liuxing1127@sina.com; c College of Chemistry, Xiangtan University Xiangtan 411105 China

## Abstract

Brominated dyes, 2C-*n* (*n* = 1–5), 3C-4 and 4C-4, were prepared through bromination of three carbazole-based D–π–A dyes, 2C, 3C and 4C with *N*-bromosuccinimide (NBS). The detailed structures of the brominated dyes were confirmed by ^1^H NMR spectroscopy and mass spectrometry (MS). The introduction of the Br atom on the 1,8-positon of carbazole moieties led to blueshifted UV-vis and photoluminescence (PL) spectra, increased initial oxidation potentials and enlarged dihedral angles, indicating bromination enhanced non-planarity of the dye molecules. In the hydrogen production experiments, with the increase of the Br content in brominated dyes, the photocatalytic activity increased continuously (except 2C-1). The dye-sensitized Pt/TiO_2,_ 2C-4@T, 3C-4@T and 4C-4@T, exhibited high hydrogen production efficiencies of 655.4, 877.9 and 905.6 μmol h^−1^ g^−1^, respectively, which were 4–6-fold higher than those of 2C@T, 3C@T and 4C@T. The enhanced performance of photocatalytic hydrogen evolution was attributed to decreased dye aggregation resulting from the highly non-planar molecular structures of the brominated dyes.

## Abstract 2

New dyes substituted by Br give excellent hydrogen production rate, 4–6 folds that for corresponding non-brominated dyes.

## Graphical abstract

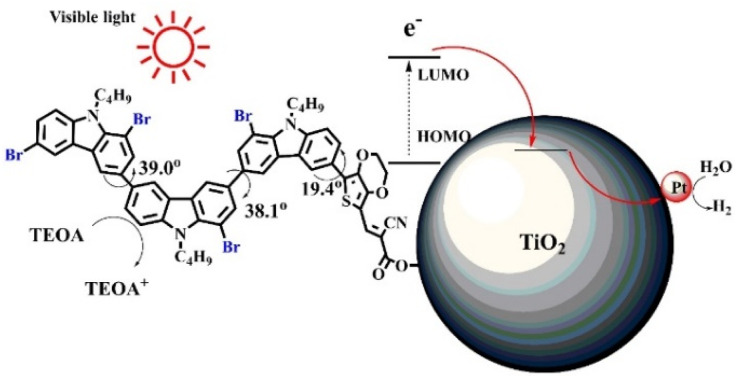

## Introduction

In recent years, solar energy has been regarded as a substitute for traditional fossil energies, such as petrol and gas. The utilization of solar energy to produce clean energy, hydrogen, is one of many important concerns in the field of renewable energy nowadays.^1,2^ Photocatalytic hydrogen evolution from water splitting using TiO_2_ as the catalyst has been proposed for nearly 50 years.^3,4^ In this process, the hydrogen evolution follows a mechanism where the excited electrons from TiO_2_ induced by light are transferred to Pt, and reduce water to hydrogen. However, because the energy gap of TiO_2_ is very wide (*ca.* 3.2 eV for anatase), it can only absorb ultraviolet light (*ca.* 4% to 6% in the solar spectrum). Many studies are devoted toward improving the light-harvesting ability of the catalyst systems.^5–8^ Among them, dyes are used for anchoring on TiO_2_ to absorb visible light, which is called dye sensitization.^9^ In addition to good light-harvesting capability, the dye-TiO_2_ system can effectively separate excited electrons and holes to avoid charge recombination. The main types of dyes include ruthenium complexes,^10–12^ porphyrins,^13^ xanthenes^14,15^ and metal-free organic compounds. Due to their designability and accessibility, metal-free organic compounds have attracted great attention in recent decades.^16–18^

A metal-free organic compound usually has a D–A or D–π–A conjugated structure. The electronic push–pull effect between the donor (D) and acceptor (A) in the structure is not only conducive to broadening the light absorption range, but also beneficial to electron–hole separation and charge transfer. When designing the structure of D–π–A conjugated dyes for photocatalytic hydrogen evolution, researchers mainly focus on three aspects: (i) donors, which are moieties including triphenylamine,^19–21^ phenothiazine,^22,23^ coumarin^24^ and carbazole^25,26^ with strong electron-donating properties and the ability to adjust the energy level and broaden the light absorption range; (ii) π link (spacer)^27–30^ which can accelerate electron transfer from D to A; (iii) side chains,^31–34^ which can control the combination mode of dyes in the dye-TiO_2_ system by adjusting hydrophilicity and steric hindrance.

Metal-free organic compounds are also used in dye-sensitized solar cells (DSSCs), which have almost been replaced by perovskite solar cells now. In the research on DSSCs, dye aggregation is considered one important factor, which usually greatly affects the photoelectric performances of DSSCs. The typical method to inhibit dye aggregation is to add an anti-aggregation agent, chenodeoxycholic acid (CDCA), into the electrolyte.^35–37^ By contrast, strategies to prevent dye molecules from aggregation by intrinsic molecular structure design are favoured by scientists, including (i) introducing alkyl chains^37–40^ and (ii) incorporating a non-planar bulky aromatic skeleton into dye molecules.^41–46^ The photovoltaic performance of DSSC was significantly improved by the inhibition of dye aggregation due to steric hindrance of alkyl chains and non-planar structures. Regarding dye-sensitized photocatalytic hydrogen evolution, inhibition of dye aggregation has also been proved to greatly improve hydrogen production efficiency.^47^

**Scheme 1:**
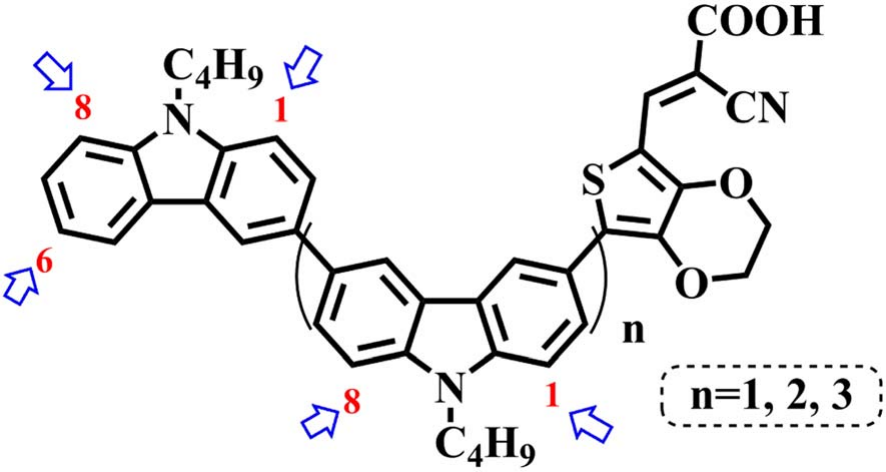
Possible bromination positions on 2C, 3C and 4C.

In our previous works, we reported three carbazole-based D–π–A dyes, 2C, 3C and 4C, and their application for DSSC^48^ and photocatalytic hydrogen evolution.^49^ In those systems, benefitting from large dihedral angles between carbazole moieties in dye molecules, dye aggregation was decreased and relatively good photocatalytic performance was afforded. Herein, we report a strategy for further change of the planarity of dye molecules by introducing Br into the 1 or 8 positions of carbazole moieties through bromination with NBS, as shown in Scheme 1. The introduction of Br can change the dihedral angles, thus increasing the non-planarity and effectively preventing dye aggregation. The enhanced non-planarity of dyes should also have great influence on their optical and electrochemical properties, and more importantly, their hydrogen production performance.

## Experimental section

### Materials

Three multi-carbazole-based aldehydes (2C–CHO, 3C–CHO, and 4C–CHO) were synthesized mainly through Suzuki coupling and the Knoevenagel reaction from carbazole. The three acids (2C, 3C and 4C) were prepared from the aldehydes as described in our previous work.^48^ NBS, cyanoacetic acid and piperidine were used as received. Platinized TiO_2_ nanoparticles (0.5 wt% Pt/TiO_2_) were prepared using a photoreduction method reported in our previous work.^50^ The sacrificial electron donor was aqueous triethanolamine (TEOA, 10 vol%) neutralized with HCl aqueous solution.

### Synthetic procedures

#### Synthesis of brominated aldehyde compounds

2C–CHO (0.306 g, 0.5 mmol) and a predetermined amount of NBS were added to DMF (30 mL) and cooled using an ice bath The reaction mixtures were stirred at ambient temperature for 24 hours in the dark. After extraction with dichloromethane, the organic layers were washed with water, dried over anhydrous sodium sulphate and concentrated under reduced pressure. The residues were purified by column chromatography (petroleum ether : CHCl_3_ : ethyl acetate = 10 : 4 : 1 in volume). The brominated products from 2C–CHO were named 2C–CHO-*n*, where *n* denotes the sample with different feeding molar ratios (*n* (NBS) : *n* (2C–CHO) = 1–5).

The aldehydes, 3C–CHO and 4C–CHO, were reacted with 4 equivalent (eq.) NBS, respectively. The corresponding brominated products obtained were named 3C–CHO-4 and 4C–CHO-4. Except that 2C–CHO-1 is a pure compound, other brominated aldehyde compounds are mixtures, with yields of 20–60%.

#### Synthesis of brominated acid compounds

The brominated aldehyde compounds (100 mg) and cyanoacetic acid (100 mg, about 10 eq.) were dissolved in CHCl_3_/acetonitrile (10 mL/10 mL), and then piperidine (0.5 mL) was dropped in. The reaction solutions were stirred at reflux temperature for 12 hours. After cooling, dilute hydrochloric acid was added for neutralization. The organic layers were separated, dried with anhydrous sodium sulphate, and concentrated under reduced pressure. The residues were separated by column chromatography (THF : MeOH = 10 : 1 in volume) to produce red powders. The obtained brominated acid compounds were named 2C-*n*, 3C-4 and 4C-4 except for 2C-1, the brominated acid compounds obtained were all mixtures, and the yield was 60–80%.

### Measurements

^1^H NMR spectra were recorded on a 500 MHz Bruker Avance III HD spectrometer using CDCl_3_ or DMSO-*d*6 as the solvent. FT-IR spectra of the samples were measured on an IRPrestige-21 instrument (Shimadzu, Japan) by a transmission method using the KBr pellet technique. Matrix assisted laser desorption ionization time-of-flight mass spectrometry (MALDI-TOF MS) was performed on a Bruker autoflflex III (Bruker, Germany). Cyclic voltammetry (CV) measurements were performed with a CHI650E electrochemical workstation. The redox potentials of the dyes were measured in a DMF solution (3 × 10^−4^ M) containing 0.1 M tetrabutylammonium hexafluorophosphate (TBAPF6) as the supporting electrolyte at a scan rate of 100 mV s^−1^, using AgCl/Ag and platinum wire as reference and counter electrodes, respectively. All potentials were calibrated with ferrocene/ferrocenium (Fc/Fc^+^) as an internal standard. UV-vis absorption spectra were collected using a UV-2550 spectrophotometer (Shimadzu, Japan). Photoluminescence (PL) and time-resolved fluorescence decay curves of the dyes in DMF solution (1 × 10^−5^ M) were obtained on a spectrophotometer of U-3310 (Hitachi, Japan).

#### H_2_ evolution measurement

In a Pyrex bottle, Pt/TiO_2_ (0.100 g) was dispersed in DMF (9.8 mL) and stirred under ultrasound for 20 min. Then, the organic dye solution (0.2 mL, 2 × 10^−3^ mol L^−1^ in DMF) was added and stirred for another 10 min to allow the organic dyes to adsorb on the catalyst. Neutralized TEOA (90 mL, 10 vol%) was added into the Pyrex bottle, and the suspension was stirred in an ultrasonic bath for 5 min and purged with N_2_ for another 30 min to remove O_2_. The Pyrex bottle containing the suspension was placed under the irradiation of a 250 W halogen lamp. The amount of evolved hydrogen was quantified using a gas chromatograph (TCD, 13X molecular sieve column) with N_2_ as the carrier gas and normalized to the 1 g of the photocatalyst.

## Results and discussion

### Synthesis and structures of brominated dyes

**Scheme 2:**
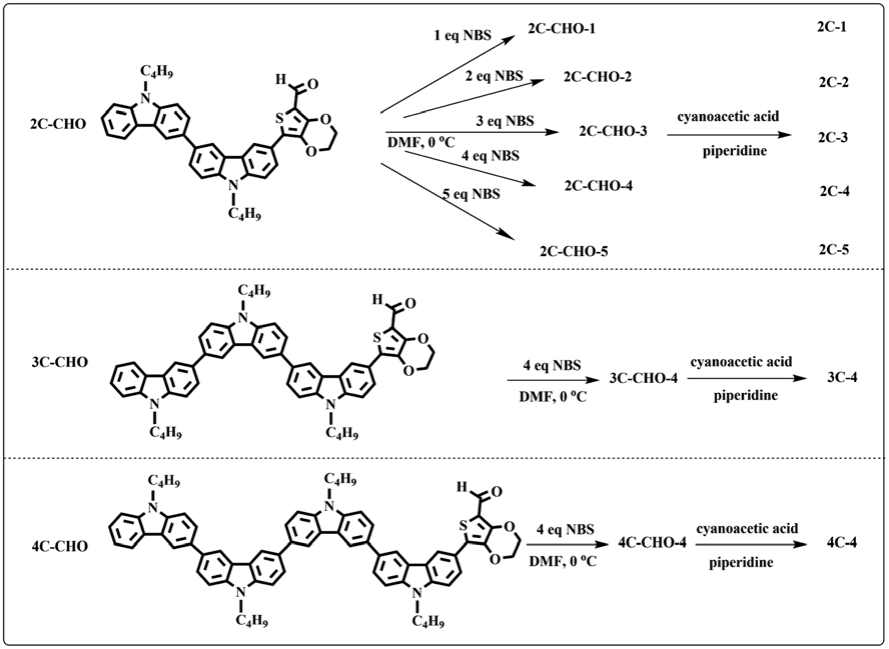
Preparation routes for brominated dyes.

All brominated dyes were prepared by bromination and Knoevenagel reactions, as shown in Scheme 2. In the bromination reaction, the aldehyde compounds, 2C–CHO, 3C–CHO, and 4C–CHO were reacted with NBS. The position and number of Br in the molecule greatly depended on the amount of NBS. When NBS was 1 eq. to 2C–CHO, the reaction was easy to perform, and the yield was high due to the high activity and selectivity of the 6-position of carbazole moiety in 2C–CHO. When the dosage of NBS was further increased, the substitution has to take place at the 1 and 8 positions. Because of large steric hindrances, further bromination at 1 and 8 positions was difficult and uncontrollable. In addition, unfavorable oxidation of the thiophene group in 2C–CHO by NBS took place, resulting in a lower number of Br in the products than the theoretical values. The oxidation became more intense as the dosage of NBS increased. When the amount of NBS was greater than 5 eq. to 2C–CHO, the yield of the brominated product was extremely low. Therefore, 2C–CHO-*n* (*n* = 1–5), 3C–CHO-4 and 4C–CHO-4 were prepared. Then, Knoevenagel reactions between the brominated aldehyde compounds and cyanoacetic acid were carried out and gave the corresponding brominated acid compounds, 2C-*n* (*n* = 1–5), 3C-4, and 4C-4.

**Fig. 1:**
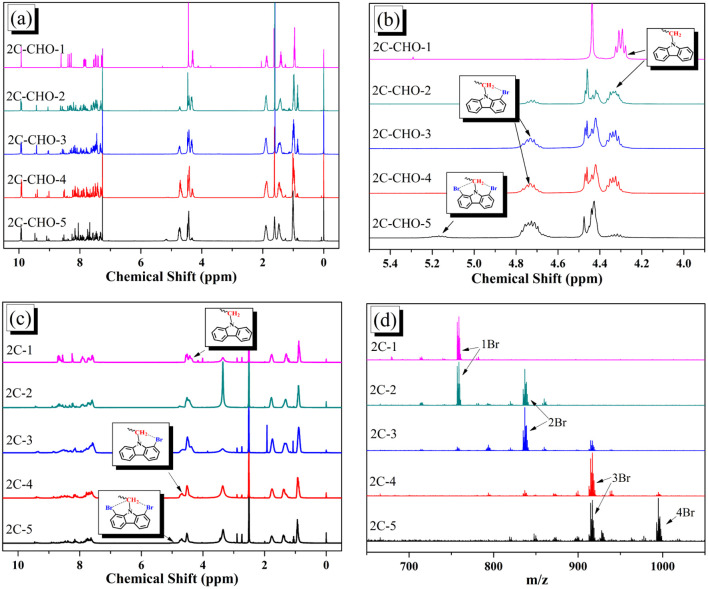
(a) Full and (b) part of ^1^H NMR spectra of 2C–CHO-n (CDCl_3_); (c) ^1^H NMR spectra of 2C-*n* (DMSO-*d*6); (d) MALDI-TOF MS spectra of 2C-*n*.

The structures of brominated aldehydes were determined by ^1^H NMR spectroscopy. It can be seen from the ^1^H NMR spectrum of 2C–CHO-1 in Fig. 1a that the peak at 9.92 ppm is assigned to CHO, and 7.25–8.62 ppm to the aromatic H on carbazole moieties, 4.42 ppm to the H on ethylidene of EDOT, 0.97, 1.43, 1.90 and 4.34 ppm to the H on the butyl chains. These results suggest that 2C–CHO-1 is a pure 6-position Br-substituted product. Unlike 2C–CHO-1, other aldehyde compounds 2C–CHO-*n* (*n* > 1) displayed very disordered peaks at 7–10 ppm, which are ascribed to aromatic H on carbazole moieties, indicating that they are mixtures. However, the peak ascribed to the H on ethylene of EDOT remains located at about 4.42 ppm. Due to the influence of *o*-Br, the peak assigned to H in –N–CH_2_– is shifted from 4.34 ppm to 4.74 ppm. In the case of 2C-CHO-5, some H in –N–CH_2_– are correlated with two *o*-Br, making the peak further move to 5.17 ppm (see Fig. 1b). This change can also be found when comparing ^1^H NMR spectra of *N*-butylcarbazole with its mono-, di-, tri- and tetra- Br-substituted compounds (Fig. S1†). Therefore, it can be concluded that as the amount of NBS increases, the number of Br on the aldehyde compounds continues to increase despite somehow deviating from theoretical calculation due to oxidation.

The resolution of ^1^H NMR spectra of brominated acid compounds is poor (see Fig. 1c), so mass spectrometry was used for more accurate analysis. As shown in Fig. 1d, the molecular peaks of 2C-1 are at 758 corresponding to one-Br-substituted compound (1Br); 2C-2 has two series of peaks, one at 758 corresponding to 1Br and another at 836 corresponding to two-Br-substituted compound (2Br); 2C-3 mainly contains peaks at 836 for 2Br and at 914 corresponding to three-Br-substituted compound (3Br); 2C-4 mainly contains peaks at 914 for 3Br; 2C-5 mainly contains peaks at 914 corresponding to 3Br and at 994 corresponding to four-Br-substituted compound (4Br). Therefore, the compositions of 2C-*n* can be inferred, as shown in Table 1. Also, from ^1^H NMR spectra and mass spectrometry (see Fig. S2 and S3†), 3C-4 and 4C-4 are found to mainly contain 3Br and 4Br, as shown in Table 1. It must be noted, however, that the molecular formula given in Table 1 is presumably obtained from mass spectrometry. Except for 2C-1, the substitution position of Br is not necessarily as shown in Table 1. Without selectivity in the bromination reactions, the brominated acid compounds except 2C-1 might contain a variety of homologues with different Br substitution positions. However, considering the steric hindrance and electronic effect, the molecular structure of each component of brominated acid compounds is described with the molecular formula shown in Table 1 for convenience.

**Table 1:** Main components in brominated dyes

Dye	Main components	
2C-1	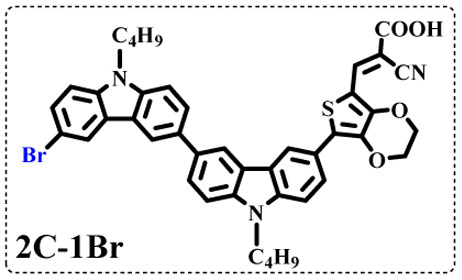	
2C-2	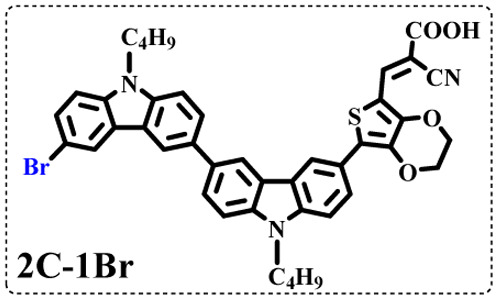	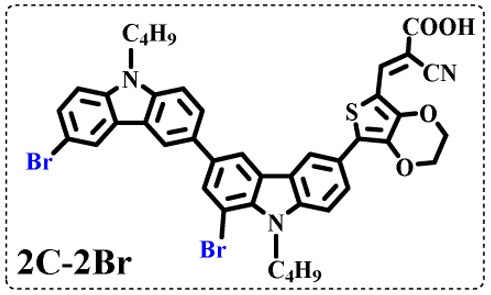
2C-3	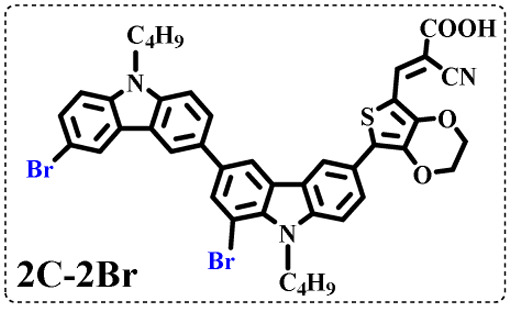	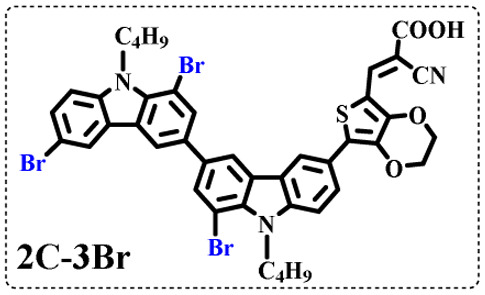
2C-4	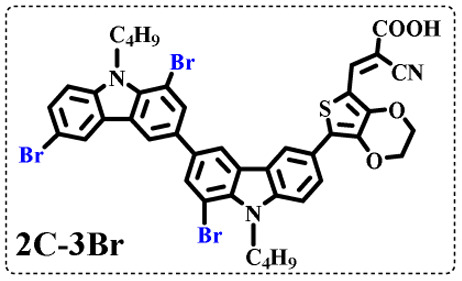	
2C-5	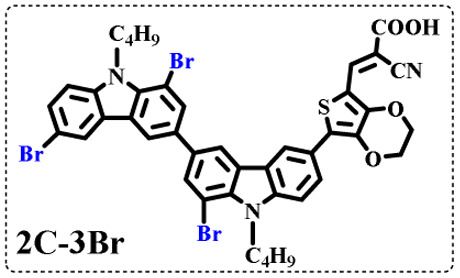	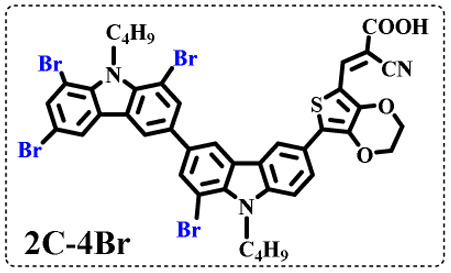
3C-4	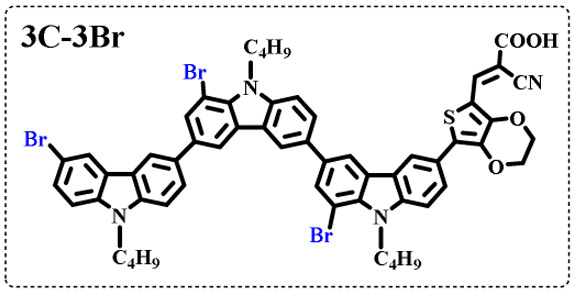	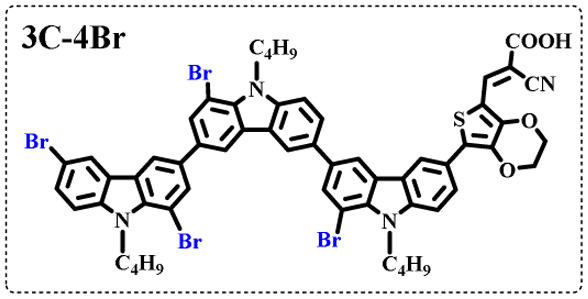
4C-4	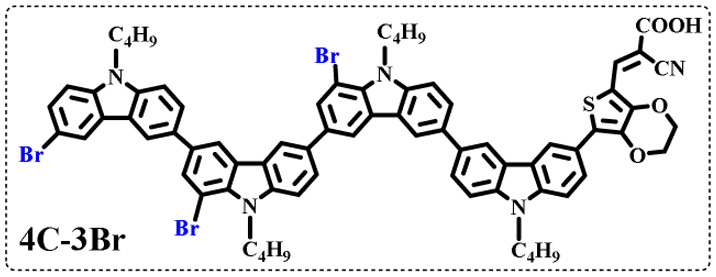	
	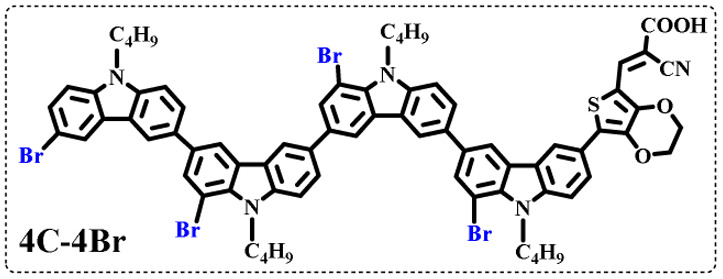	

### Optical properties

**Fig. 2:**
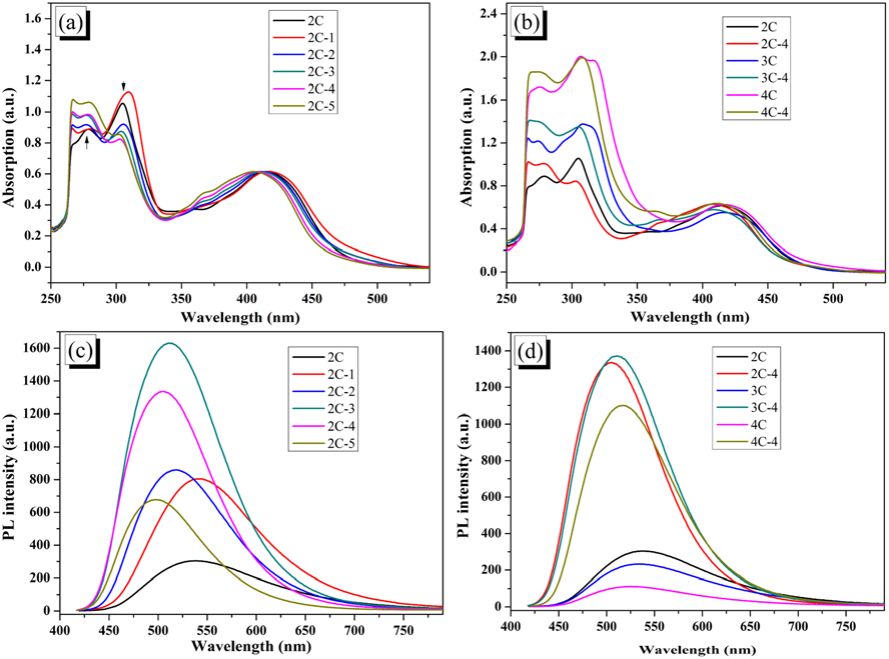
(a) UV-vis absorption spectra of 2C and 2C-*n*; (b) UV-vis absorption spectra of 2C–4C and their brominated products with 4 eq. NBS; (c) PL spectra of 2C and 2C-*n*; (d) PL spectra of 2C–4C and their brominated products with 4 eq. NBS. (All measured in DMF solution (1 × 10^−5^ M)).

The UV-vis absorption spectra of 2C–4C and all brominated dyes in DMF solution are shown in Fig. 2a and b. In Fig. 2a, compared with 2C, the peak at 350–500 nm of 2C-1 attributed to intramolecular charge transfer (ICT) shows a slight redshift, which is due to the increased conjugation degree by 6-position substitution of electron-donating Br. 2C-*n* (*n* > 1) showed a different trend from 2C-1. With the increase of *n* (*i.e.*, the increase of Br content), the peak at 350–500 nm was continuously shifted toward a low wavelength. It was also found that the peak intensity at 300–325 nm (attributed to π–π* transition of conjugated bicarbazole) kept declining while the peak intensity at 250–285 nm (attributed to π–π* transition of single carbazole) continued to increase. It is believed that the heavier bromination occurred at the 1 and 8 positions, the larger steric hindrance by Br existed, which led to increasing dihedral angles between heterocycles and a reduced conjugation degree. That is to say, bromination breaks down the conjugation, making the conjugated bicarbazole tend to be a single carbazole. As shown in Fig. 2b, the UV-vis spectra of 3C-4 and 4C-4 also have similar characteristics to that of 2C-4, with a blueshift compared with that of 3C and 4C.

The photoluminescence (PL) spectra of the dyes are shown in Fig. 2c and d. Consistent with the results of UV-vis spectra, the PL peak of 2C-1 has a small red shift relative to 2C, while 2C-*n* (*n* > 1) all show a blueshift. The wavelength of the maximum peak decreases with the increase of *n*, which also evince that the conjugation degree of dye molecules decreases in response to bromination. In addition to that, for samples testing at the same concentration, when *n* rises from 1 to 3, the PL intensity increases sharply. This result is reasonable because of the electron donation of Br and decreased dye aggregation caused by the lowered molecular planarity. When *n* continues to increase from 3 to 5, the fluorescence intensity decreases, which might be a result of the lowered conjugation degree of the heavily brominated dyes. Fig. 2d shows the change of PL spectra of 2C-4–4C-4, which have an obvious redshift compared with their non-brominated counterparts, 2C–4C.

### Electrochemical properties

**Fig. 3:**
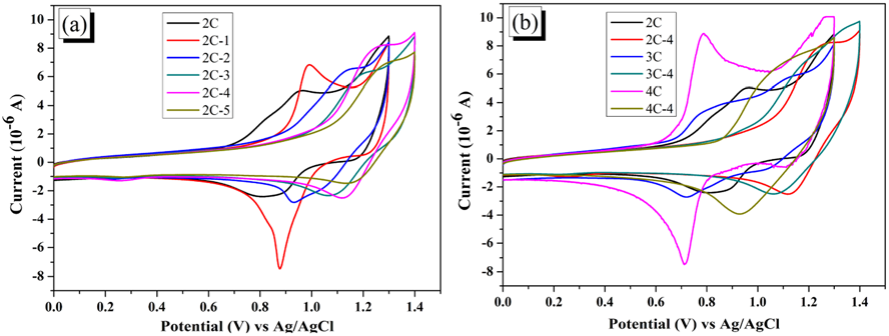
(a) CV curves of 2C and 2C-*n*; (b) CV curves of 2C–4C and their brominated products with 4 eq. NBS (all measured in DMF solution (3 × 10^−4^ M) containing 0.1 M TBAPF6 as supporting electrolyte at a scan rate of 100 mV s^−1^), HOMO levels and LUMO levels of the dyes were calculated from the initial oxidation potential and *E*_0–0_, and the results are shown in Table 2. HOMO levels of 2C–4C are around 0.94 V *versus* normal hydrogen electrode (NHE) and the LUMO levels are around −1.75 V *versus* NHE. With the increase of n, the HOMO levels of the brominated dyes 2C-*n* increase from 1.13 to 1.27 V *versus* NHE. The HOMO levels of 3C-4 and 4C-4 are 1.18 and 1.10 V *versus* NHE, respectively. The LUMO levels present a similar trend as the HOMO levels. This result indicates that the introduction of Br can adjust HOMO and LUMO levels of brominated dyes.

The electrochemical behaviors of the dyes were measured by cyclic voltammetry (CV), and the results are shown in Fig. 3. Although most brominated dyes are mixtures, it can be seen from Fig. 3a that the initial oxidation potentials of dye 2C-*n* increase constantly from 0.7 V to more than 1.0 V with the increase of *n*. From Fig. 3b, 2C–4C displayed much lower initial oxidation potentials than 2C-4–4C-4. The significant impact of the introduction of Br on the electrochemistry of dyes is the result of the decreased conjugation degree after bromination. On the other side, the redox process of dyes is reversible based on the symmetrical curve shape, indicating that the dyes have decent stability in the redox process.

**Table 2:** Absorption, emission, and electrochemical properties of the dyes

Dye	*λ*_max abs_ a (nm)	*λ*_max emi_ a (nm)	*V*_onset_ b (V)	HOMO(v)	*E*_0–0_ c (V)	LUMO^d^ (V)
2C	415	540	0.71	0.94	2.69	−1.75
2C-1	417	541	0.90	1.13	2.66	−1.53
2C-2	412	519	0.90	1.13	2.72	−1.59
2C-3	411	512	0.98	1.21	2.75	−1.54
2C-4	408	502	1.02	1.25	2.76	−1.51
2C-5	405	498	1.04	1.27	2.78	−1.51
3C	416	534	0.68	0.91	2.68	−1.77
3C-4	408	511	0.95	1.18	2.75	−1.57
4C	433	525	0.73	0.96	2.67	−1.71
4C-4	423	518	0.87	1.10	2.72	−1.62

a Measured in DMF solution (1 × 10^−5^M).

b Measured in DMF solution (3 × 10^−4^ M) using 0.1 M TBAPF6 as a supporting electrolyte, Pt wire as the working and counter electrode, and Ag/AgCl as the reference electrode, respectively.

c Calculated from the intersection between the absorption and emission spectra (*E*_0–0_ = 1240/*λ*).

d Obtained as HOMO − *E*_0–0_.

### Theoretical calculation

Density functional theory (DFT) calculations at the B3LYP/6-31G* level were carried out to further understand the geometrical configurations and electron distributions of 2C–*n*Br and tetra-brominated derivates, 3C–4Br and 4C–4Br are shown in Table 1.

For 2C and 2C–*n*Br containing two carbazole moieties, with the increase of *n*, the dihedral angle between two carbazole increases from 37.3 to 38.4° and the dihedral angle between carbazole and thiophene increased from 18.3 to 21.4° (see Fig. S4†). The increase is not very large because Br has a small atomic size, but may greatly destroy the conjugation degree of the molecule, considering the great changes in optical and electrochemical properties. 3C–4Br and 4C–4Br also exhibit larger dihedral angles than 3C and 4C, respectively.

From Table S1,† the HOMO and LUMO levels gradually enhance with the increase of *n*, just like the CV results. *E*_0–0_ increases monotonically, indicating that the introduction of Br leads to a narrow absorption range, which is also consistent with the UV-vis data of 2C-*n* (except 2C-1). In addition, the optimized structures and electric distribution in the HOMO and LUMO levels of 2C–4Br–4C–4Br are presented in Table 3. The HOMO is mainly distributed on electron-donating carbazole moieties, while the LUMO is predominantly delocalized over the cyanoacrylic acid segment and extended to thiophene moiety. This kind of electronic distribution is favorable to the effective separation of charge holes during photocatalytic hydrogen evolution.

**Table 3:** Frontier molecular orbitals of the HOMO and LUMO calculated using DFT

Dye	HOMO	LUMO
2C–4Br	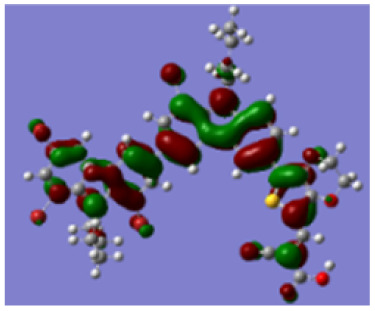	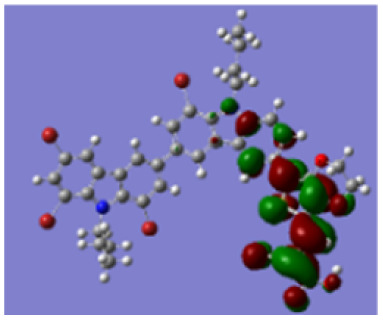
3C–4Br	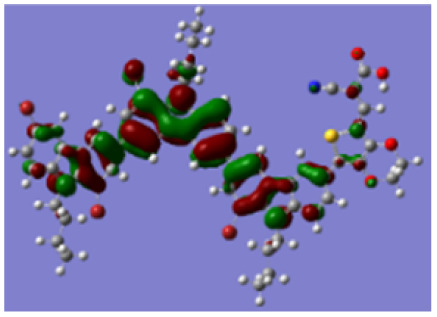	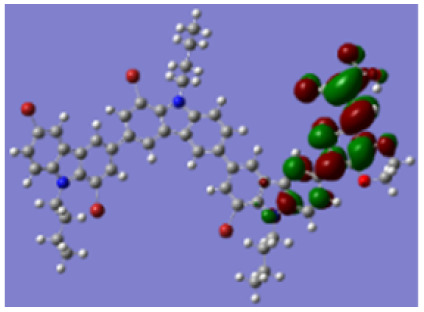
4C–4Br	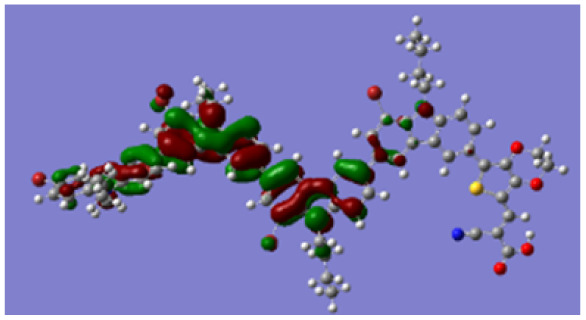	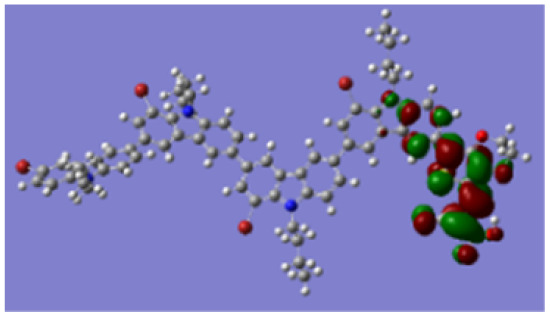

### Photocatalytic hydrogen production

The photocatalytic systems, Pt/TiO_2_ sensitized with each dye were named dye@T, for example, 3C-4@T. Without the presence of dyes or light irradiation, the hydrogen production efficiency was lower than 5 μmol h^−1^ g^−1^, demonstrating the importance of the sensitization and light absorption of these dyes.

**Fig. 4:**
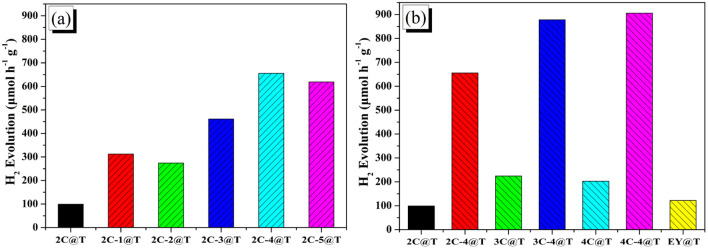
Hydrogen evolution activities of dye-sensitized Pt/TiO_2_ photocatalytic systems based on (a) 2C and 2C-*n*; (b) 2C–4C, 2C-4–4C-4, and EY.

The results of photocatalytic hydrogen evolution over 2C@T and 2C-*n*@T with 10 vol% neutralized TEOA as the sacrificial agent under visible light irradiation (*λ* ≥ 420 nm) are shown in Fig. 4a. In the first hour of illumination, the hydrogen production efficiency over 2C@T was 99.2 μmol h^−1^ g^−1^, while that over 2C-1@T increased to 321.5 μmol h^−1^ g^−1^, which is due to the wider visible light absorption range brought by Br. The hydrogen production efficiency of 2C-2@T was slightly lower than that of 2C-1@T, which is due to the narrower visible light absorption range. With the further increase of *n*, the hydrogen production efficiency over 2C-*n*@T continuously rises, from 461.3 μmol h^−1^ g^−1^ for 2C-3@T to 655.4 μmol h^−1^ g^−1^ for 2C-4@T and 618.6 μmol h^−1^ g^−1^ for 2C-5@T. Then, the hydrogen production performances of Eosin Y (EY), 2C–4C and their brominated products with 4 eq. NBS were compared together, and the results are shown in Fig. 4b. The hydrogen production efficiency over EY@T was only 122.6 μmol h^−1^ g^−1^ and those over 2C@T, 3C@T, and 4C@T were 99.2, 224.6 and 202.6 μmol h^−1^ g^−1^, respectively. The brominated dyes, 4@T, 3C-4@T, and 4C-4@T had hydrogen production performance of 655.4, 877.9, and 905.6 μmol h^−1^ g^−1^, respectively, 4–6 folds higher activity than their counterparts, 2C@T, 3C@T, and 4C@T.

The apparent quantum yields (AQY) of the selected photocatalytic system 3C-4@T were investigated and the data are listed in Table S2.† Under monochromatic light irradiation by band-pass filters, *λ* = 450, 475, 500 nm, the AQY values of 3C-4@T are 2.55%, 1.79%, and 1.64%, respectively. The photocatalytic activity can also be discerned from transient photocurrent responses, as shown in Fig. S5.† The photocurrents of 2C-4/TiO_2_, 3C-4/TiO_2,_ and 4C-4/TiO_2_ are much higher than that of nonbrominated dyes.

**Fig. 5:**
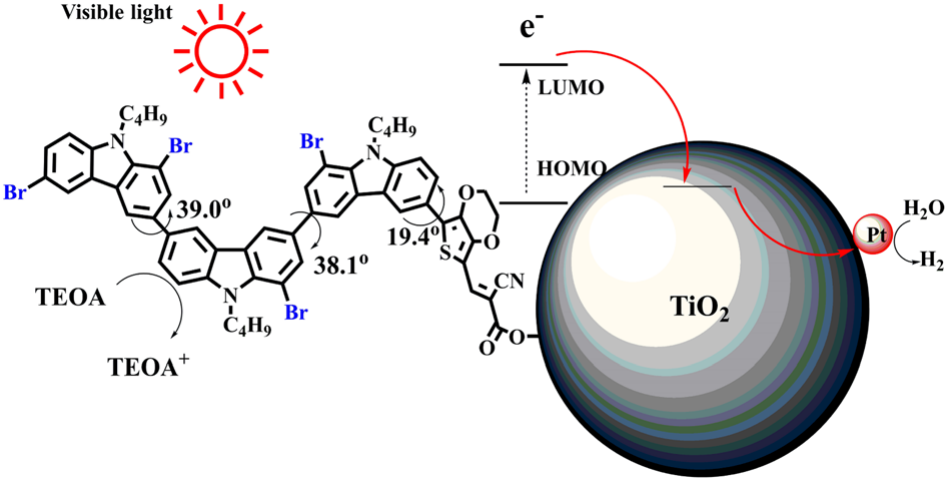
Probable mechanism of photocatalytic hydrogen evolution.

Therefore, it can be concluded that the introduction of Br into dye molecules is very effective for improving photocatalytic hydrogen evolution. Based on the above-mentioned data from UV-vis, PL, CV testing, and DFT calculations, bromination could give rise to two results, blueshift absorption spectra and enhanced non-planarity of dye molecules (excluding 2C-1). Since a narrow absorption range is harmful for utilizing visible light, non-planarity is supposed to have a close correlation with the improvement of hydrogen production performance. Enhanced non-planarity due to the introduction of Br means low dye aggregation, which is considered the reason for the strong recombination of photoinduced electron–hole pairs. When each dye molecule is not aggregated with other dye molecules and independently adsorbed on Pt/TiO_2_, as shown in Fig. 5, the energy stored in excited dye molecules after absorbing light should not easily be consumed through PL emission, and the photogenerated electron–hole can be effectively separated and transferred to Pt/TiO_2_.

**Fig. 6:**
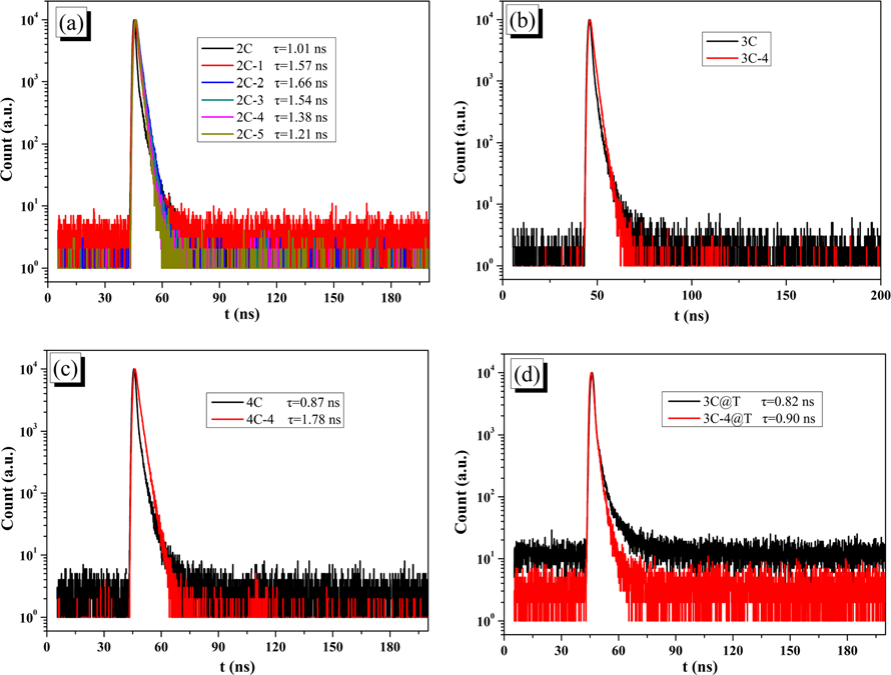
PL delay in DMF solution of (a) 2C and 2C-*n*; (b) 3C and 3C-4; (c) 4C and 4C-4; (d) 3C@T and 3C-4@T.

Transient fluorescence can obtain the fluorescence lifetime (*τ*), which can be used to analyze the electron–hole separation. The decay curves and *t* of all brominated dyes are shown in Fig. 6a–c. Brominated dyes have increased *t* compared with non-brominated dyes. Lower dye aggregation by bromination makes it difficult for excited brominated dyes to lose energy through intermolecular relaxation and thus obtain large *τ*, which can give enough time for electrons to migrate to Pt/TiO_2_.^51^ On the other hand, the decay curves and *t* of 3C@T and 3C-4@T are shown in Fig. 6d. Because of the PL quenching caused by the electron transfer from the dye to Pt/TiO_2_, the *t* of dye-sensitized Pt/TiO_2_ has inverse meaning compared with that of pure dye. Unlike the small difference between the *t* of 3C and 3C@T, 3C-4@T has a *t* of 0.90 ns, much lower than the 1.66 ns of 3C-4. This indicates that the electron transfer from 3C-4 to Pt/TiO_2_ is very fast and correspondingly charge recombination is reduced.^52,53^

**Fig. 7:**
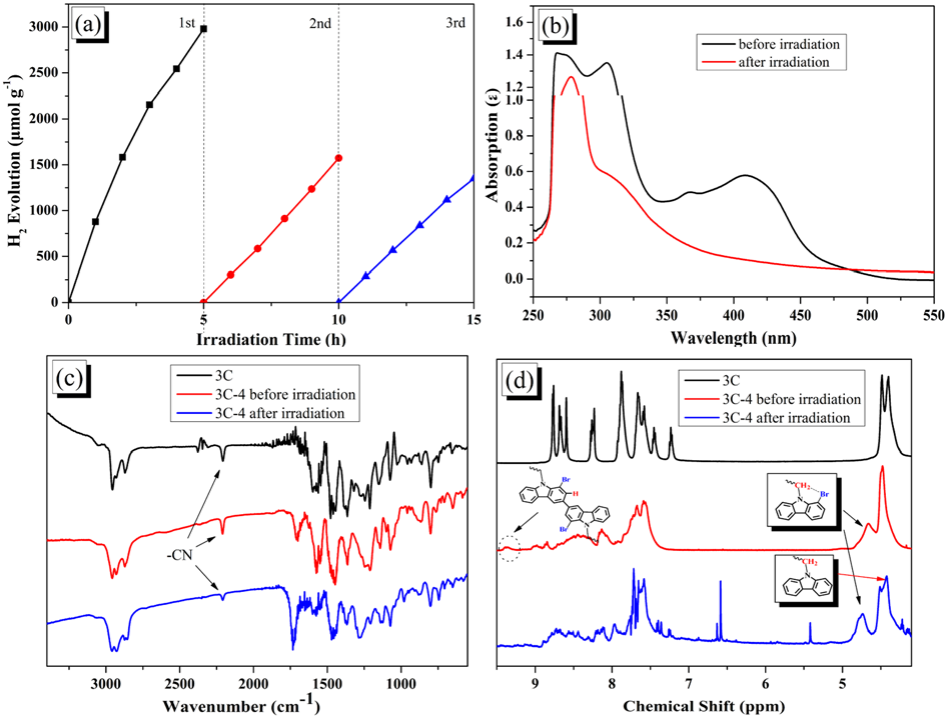
(a) Cycling measurements of hydrogen evolution activities of 3C-4@T; (b) UV-vis spectra of 3C-4 before and after irradiation; (c) FT-IR spectra of 3C, 3C-4 before and after irradiation; (d) ^1^H NMR spectra of 3C, 3C-4 before and after irradiation.

The stability of photocatalytic hydrogen evolution for 3C-4@T was investigated. As shown in Fig. 7a, with the increase of irradiation time, the activity of hydrogen production decreased significantly. By comparing the absorption spectra of 3C-4 before and after hydrogen production (see Fig. 7b), it was found that the peak at 350–500 nm attributed to ICT disappeared after irradiation due to the instability of the cyanoacrylic acid segment.^49,54^ The degradation of cyanoacrylic acid segments under light can be confirmed from the decreased absorption peak at 2210 cm^−1^ (ascribed to CN groups) in Fig. 7c. In addition, according to the study of EY, there may also be debromination in the presence of Pt and hydrogen. Because of the debromination, the disappearance of the peaks at 9.3 ppm and the change of the peaks at 4.4–4.8 ppm was found in Fig. 7d after light irradiation.

This indicates that the molecular structure of the brominated dyes needs further improvement. Therefore, we have now started to use arylboronic esters to react with Br on brominated dye molecules. Furthermore, vulnerable cyanoacrylic acid segments will be replaced with carboxyl acid segments. By strengthening the non-planarity with starburst aryl groups and using durable stable carboxyl acid segments, new 1,8-aryl-substituted dyes should obtain highly stable and effective hydrogen production activity.

## Conclusions

In summary, a series of brominated carbazole-based D–π–A organic dyes were synthesized and served as sensitizers for photocatalytic hydrogen evolution. With the increase of bromination degree, the brominated dyes showed a blueshift of UV-vis absorption and PL spectra (except 2C-1), and an increase of HOMO and LUMO levels, indicating that the substitution at the 1- and 8-positions by Br decreased the molecular planarity. The brominated dyes exhibited much higher activity than the original 2C, 3C and 4C. The dye-sensitized Pt/TiO_2_ photocatalytic systems, 2C-4@T, 3C-4@T and 4C-4@T, displayed great hydrogen production efficiencies of 655.4, 877.9 and 905.6 μmol h^−1^ g^−1^, respectively, a 4–6-fold enhancement compared to those of photocatalytic systems based on corresponding non-brominated dyes. The inhibition of dye aggregation, which is due to the low molecular planarity caused by the introduction of Br, leads to the higher photocatalytic efficiency. Although these brominated dyes have poor stability, our findings can provide useful ideas for dye design for photocatalytic hydrogen evolution.

## Author contributions

Zhangli Hu, Jiamin Kuang, and Wenmo Fu contributed equally to conceptualization, methodology, and data curation. Longxin Hu: data curation. Hua Lai: writing – review and editing. Huanian Zhang: software, formal analysis. Xing Liu: supervision.

## Conflicts of interest

The authors declare no competing financial interest.

## Supplementary Material

RA-013-D3RA02785F-s001
