# Supplementary material for: Impact of the bromination of carbazole-based D–π–A organic dyes on their optical and electrochemical properties and visible-light-driven hydrogen evolution
Source: RSC Adv. 2023 Jul 4;13(29):20031–9. doi: 10.1039/d3ra02785f (PMC10318484; doi:10.1039/d3ra02785f)
Supplement: RA-013-D3RA02785F-s001 [file RA-013-D3RA02785F-s001.pdf]

Electronic Supplementary Material (ESI) for RSC Advances.
This journal is © The Royal Society of Chemistry 2023

Supporting Information for

"Impact of bromination of carbazole-based D-π-A organic dyes on their photoelectrochemical properties and visible light-driven hydrogen evolution"

Zhangli Hu,[a] Jiamin Kuang,[a] Wenmo Fu,[a] Longxin Hu,[a] Hua Lai, *[a,b] Huanian Zhang[c] and Xing Liu*[a,b]

[a]College of Chemistry Materials, Hengyang Normal University, Hengyang, 421008, China.

[b]Hunan Provincial Key Laboratory of Functional Metal-Organic Compounds, Hengyang, 421008, China.

[c]College of Chemistry, Xiangtan University, Xiangtan, 411105, China.

1. Influence of bromination on the chemical shift of -*N*-CH_2-

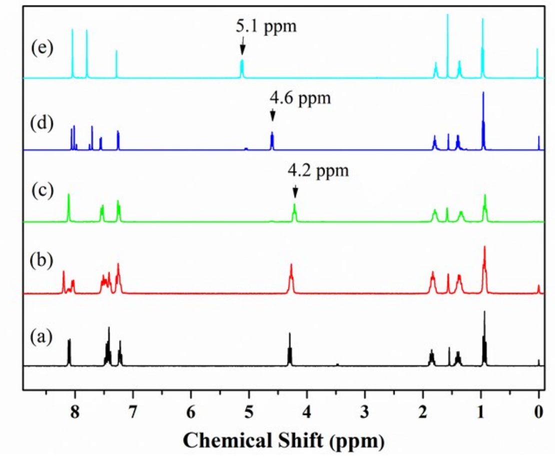

Fig.S1 ^{1}H NMR spectra of (a) *N*-butylcarbazole; (b) 3-Br-*N*-butylcarbazole; (c)3,6-di-Br-*N*-butylcarbazole;(d)1,3,6-tri-Br-*N*-butylcarbazole; (e) 1,3,6,8-tetra-Br-*N*-butylcarbazlole)

2.^{1}H NMR and MALDI-TOF MS spectra

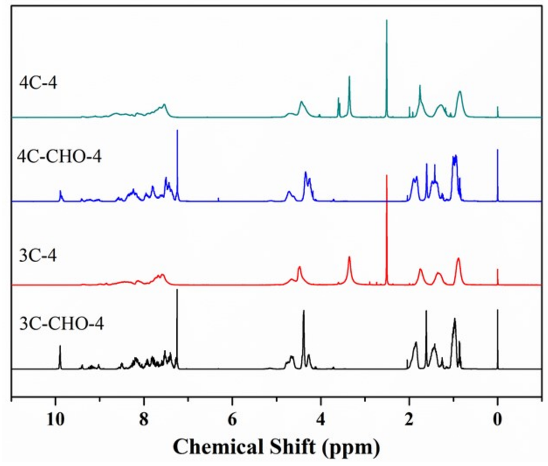

Fig.S2 ^{1}H NMR spectra of 3C-CHO-4, 3C-4、4C-CHO-4 and 4C-4

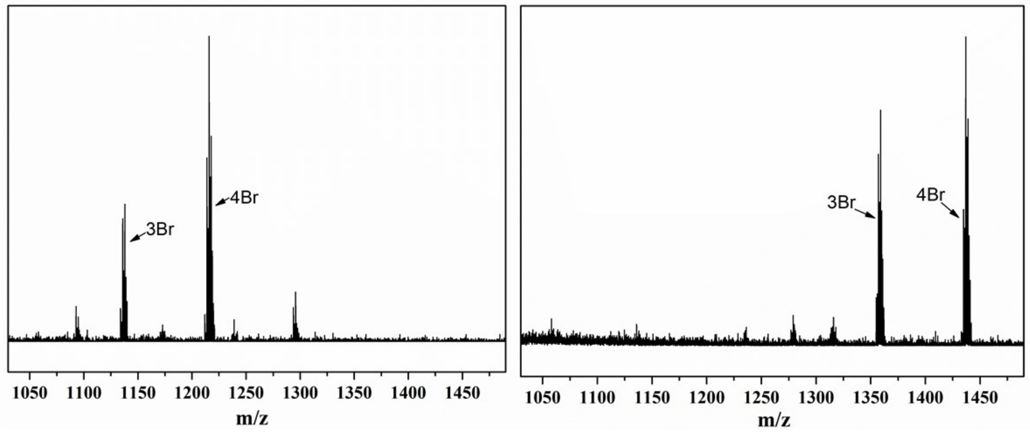

Fig.S3 MALDI-TOF MS spectra of 3C-4（left）and 4C-4（right）

3.Theoretical calculation

3.1 Dihedral angles

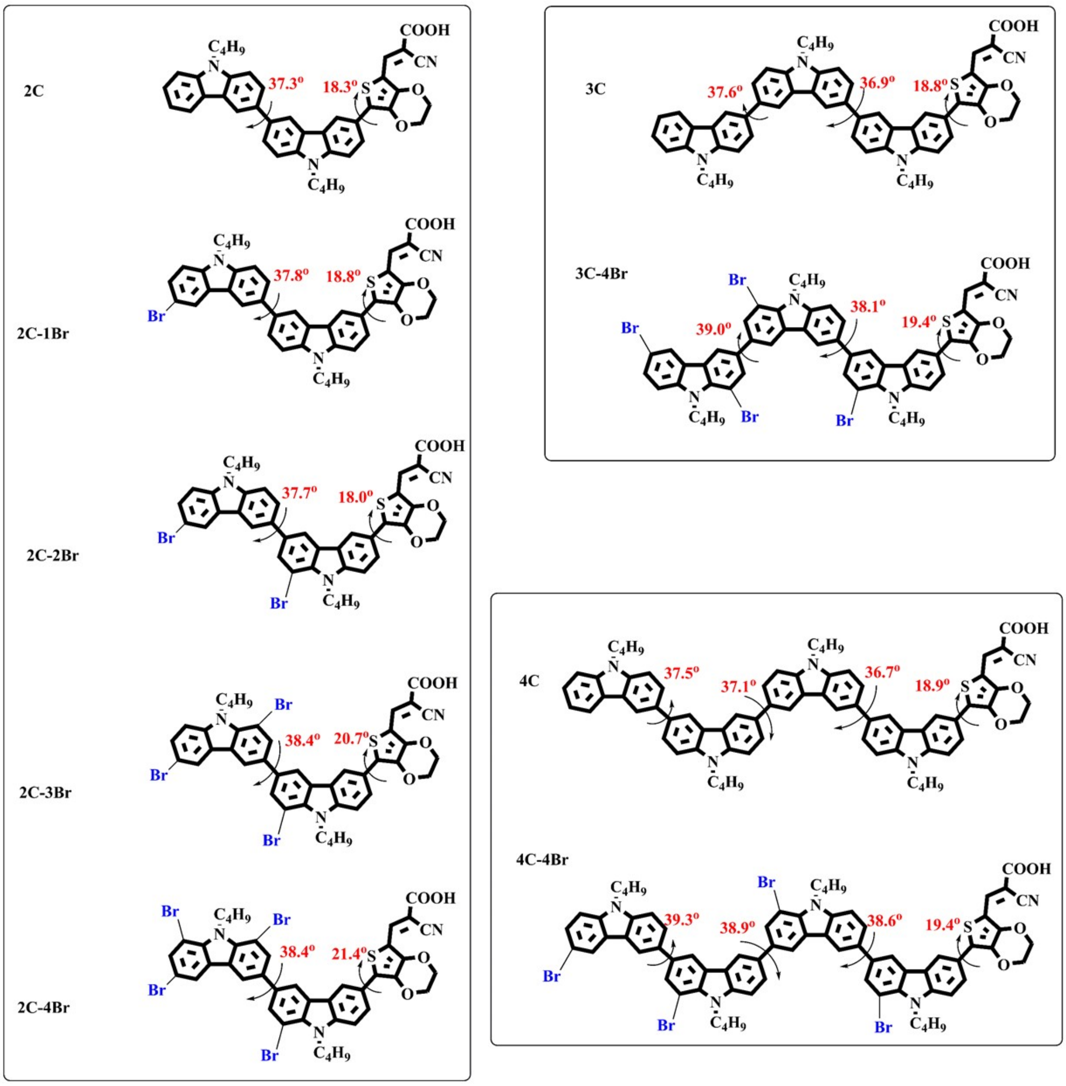

Fig.S4 Dihedral angles between heteroaromatic cycles in brominated dyes

3.2 HOMO and LUMO levels

Table S1 Calculated HOMO and LUMO levels of dyes

Dye	HOMO (eV)	LUMO (eV)	E_{0-0} (eV)
2C	-2.42	-5.00	2.58
2C-1Br	-2.46	-5.12	2.67
2C-2Br	-2.53	-5.23	2.70
2C-3Br	-2.54	-5.34	2.80
2C-4Br	-2.57	-5.42	2.85
3C	-2.41	-4.82	2.41
3C-4Br	-2.53	-5.18	2.64
4C	-2.41	-4.74	2.32
4C-4Br	-2.53	-5.03	2.50

4.Transient photocurrent responses

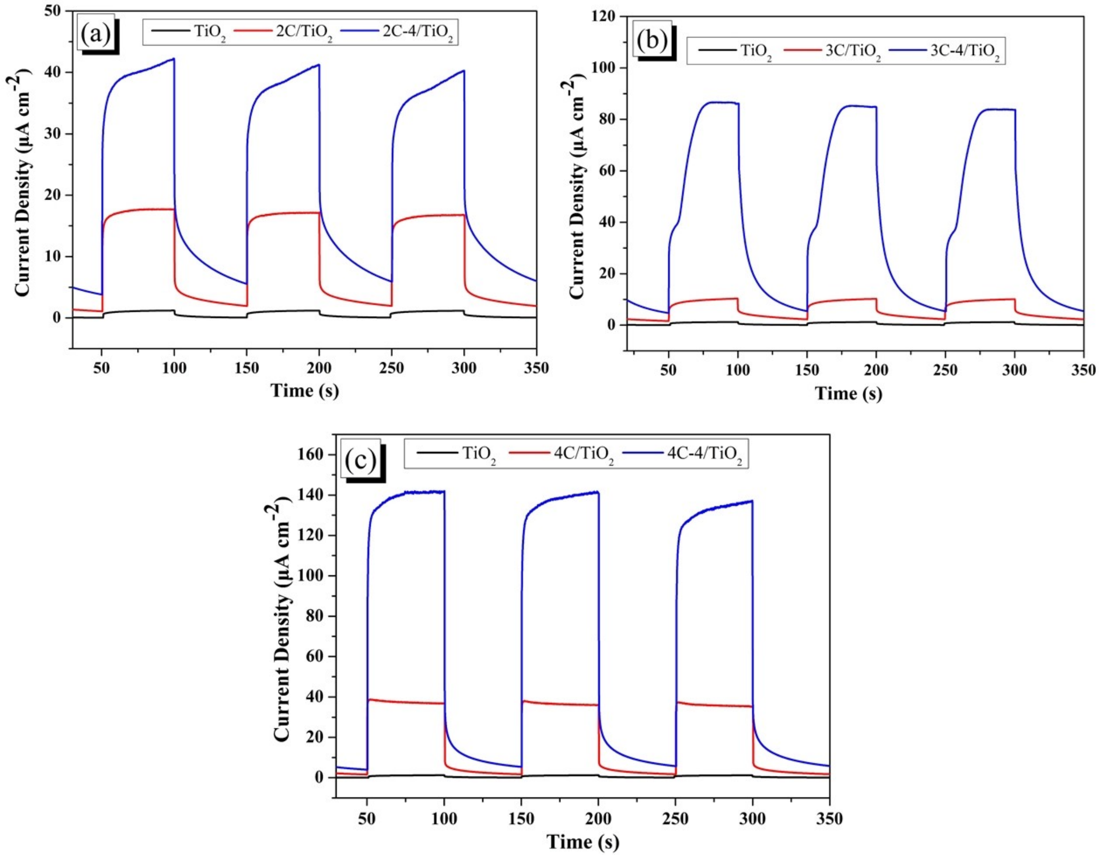

Fig.S5 Transient photocurrent densities of TiO_2 and dye-sensitized TiO_2 in 0.1 M Na_2SO_4 TEOA solution under visible light irradiation.

5. Apparent quantum yields (AQY) *

Table S2 Calculated AQY of 3C-4 under monochromatic light irradiation

Wavelength (nm)	Light intensity (mW/cm^{-2})	Amount of H_2 (μmol)	AQY
450	37	3.96	2.58
475	82	2.55	2.67
500	59	2.62	2.70

*AQY data were calculated according to the method from the literature (*J. Mater. Chem. C*, 2020,8, 14864-14872)
